# Prevalence, species distribution, and risk factors of fungal colonization and infection in patients at a burn intensive care unit in Vietnam

**DOI:** 10.18502/cmm.6.3.4664

**Published:** 2020-09

**Authors:** Be Nguyen Van Bang, Nguyen Thanh Xuan, Dinh Xuan Quang, Cao Ba Loi, Nguyen Thai Ngoc Minh, Nguyen Nhu Lam, Do Ngoc Anh, Truong Thi Thu Hien, Hoang Xuan Su, Le Tran-Anh

**Affiliations:** 1 Department of Hamatology, Toxicology, Radiation, and Occupational Diseases, Military Hospital 103, Vietnam Military Medical University, Ha Dong, Ha Noi, Vietnam; 2 Department of Medical Education, Military Hospital 103, Vietnam Military Medical University, Ha Dong, Ha Noi, Vietnam; 3 Department of Scientific and Training Management, National Institute of Malaria, Parasitology, and Entomology, Nam Tu Liem, Ha Noi, Vietnam; 4 Intensive Care Unit, National Hospital of Burn, Vietnam Military Medical University, Ha Dong, Ha Noi, Vietnam; 5 Department of Parasitology, Vietnam Military Medical University, Ha Dong, Ha Noi, Vietnam; 6 Department of Microbiology, National Hospital of Burn, Vietnam Military Medical University, Ha Dong, Ha Noi, Vietnam; 7 Department of Microbiology and Pathogens, Institute of Biomedicine and Pharmacy, National Hospital of Burn, Vietnam Military Medical University, Ha Dong, Ha Noi, Vietnam These authors contributed equally to this work and acted as joint first authors

**Keywords:** Fungal colonization, Fungal infection, Burn patients, Intensive care unit, Vietnam

## Abstract

**Background and Purpose:**

Burn patients are at a higher risk of infections caused by different organisms. This study aimed to address the prevalence, causative species, and factors related to fungal colonization or infection in patients with acute severe injuries admitted to the intensive care unit (ICU) of a burn hospital in northern Vietnam.

**Materials and Methods:**

This prospective study was conducted on 400 patients in a burn ICU between 2017 and 2019. Clinical samples were weekly collected and screened for fungi, and relevant clinical information was obtained from medical records.

**Results:**

According to the results, 90% of the patients were colonized with fungi. Out of this group, 12.75% of the cases had invasive fungal infection (IFI). Eleven yeasts and six mold species were isolated from the patients, with the most common species being *Candida tropicalis* (45.56%) and *C. albicans* (41.94%). Among the eleven species causing fungal wound infection (FWI), the most common agents were *Candida* (66.7% of FWI patients) and *Aspergillus* (38.5%) species. Three *Candida* species isolated from blood were *C. tropicalis* (66.7%), *C. albicans* (20.0%), and *C. parapsilosis* (14.3%). No factors were found to expose the patients to a higher risk of fungal colonization. However, hyperglycemia, prolonged ICU stay, and heavy *Candida* species colonization were found to be independently predictive of IFI.

**Conclusion:**

Burn patients are at the risk of fungal infection with *Candida* species (especially *C. tropicalis*) and *Aspergillus* as the most frequently responsible agents. Continuous surveillance of fungi and appropriate management of pathophysiological consequences are essential to prevent fungal infection in burn patients.

## Introduction

Burn injuries are among the most common and detrimental types of all injuries and account for 180,000 deaths annually [ 1 ]. The leading cause of mortality among burned patients is infection (75%) [ 2 ]. Burn wounds provide an ideal port of entry for organisms and induce substantial immune dysfunction [ 3 ]. Accordingly, burn patients are at a higher risk of developing infections, compared with other hospitalized patients [ 4 ]. The most common agents of infection among burn patients are bacteria and fungi [ 2 , 5 ]. Due to the better care of burn patients and availability of effective antibiotics, there is a decreasing trend in infection due to drug- sensitive bacteria. However, the number of infections caused by multi-drug resistant bacteria or fungi is on an increasing trend [ 5 , 6 ].

Fungal infections usually occur after a period of colonization with fungal strains either from hospital environment or patients [ 7 - 10 ]. The eﬀective prevention and treatment approaches of fungal infection should be based on updated and local epidemiological data because the prevalence, species distribution, and factors related to colonization or infection vary from time to time and place to place [ 11 ].

Vietnam is a developing country; regarding this, people living there are at a higher risk of burn injuries, compared to those living in developed countries [ 12 ]. However, no data on fungal colonization and infection among burn patients in Vietnam have been published. Regarding this, the aim of the present study was to address the prevalence, causative species, and factors related to fungal colonization or infection in patients with acute severe injuries admitted to the intensive care unit (ICU) of a burn hospital in northern Vietnam.

## Materials and Methods

**Ethics statement**

The study was approved by the Ethics Committee of Vietnam National Institute of Malariology, Parasitology and Entomology (Decision number 1172/QD-VSR, 28^th^ October 2016). Written informed consent was obtained for participation in the study. In cases where patients were not able to give consent themselves, the consent was obtained from their legal representatives.

**Patients and data collection**

The current investigation was conducted in the National Hospital of Burn (NHB), a teaching hospital of Vietnam Military Medical University (VMMU) during January 2017 and December 2019. Agent isolation and species identification were performed in the laboratories of the Department of Microbiology, NHB and Department of Parasitology, VMMU. All patients in the ICU of NHB during 2017-2019 who agreed to participate in this prospective study were enrolled in the research. The inclusion criteria were admission within 7 days of injury and presence of a thermal burn.

Burn injuries were managed at the NHB according to standard care for severe burn patients, such as aggressive resuscitation, use of silver sulfadiazine cream, wet wound dressing, surgical excision, and empirical antibiotic therapy. The data were obtained from inpatient charts and electronic medical records using a standardized case report form. Data collection was performed by senior clinicians who were blind to the study purpose and design. The Wallace rule was used to estimate the percentage of the total body surface area burned (% TBSA) and full-thickness surface area of patients [ 13 ]. The acute physiology and chronic health evaluation (APACHE) was used upon ICU admission to assess the injury severity for each patient [ 14 ].

**Fungal surveillance**

Non-sterile samples (i.e., mouth swabs, rectum swabs/feces, wound swabs, urine, and tracheal aspirates) were collected weekly to detect the presence of microorganisms as part of the hospital standard of care. Wound biopsy and blood were taken in cases with clinically suspected fungal wound infection (FWI) (presence of pus, increased exudation, leathery appearance, change in wound color, inflammation, or graft lysis) or fungemia [ 2 , 15 ]. The non-sterile samples were taken by two ready-made sterile cotton swabs. Blood was collected from a clean, unburnt site following routine protocols. Furthermore, biopsy (a 0.5×0.5-cm full-thickness sample) was taken from the most apparently infected site of the wound up to the healthy layer and then transferred to the laboratory in normal saline and 10% formaldehyde solution.

Fluids and secretions were microscopically examined after being processed by 10% potassium hydroxide. The biopsy samples fixed in 10% formaldehyde solution were sent to the pathology laboratory for hematoxylin and eosin (H&E) and periodic acid shift (PAS) staining, followed by an examination by pathologists. Non-sterile and tissue samples were cultured onto Sabouraud dextrose agar (SDA, BioMerieux, France) and slants containing gentamicin (0.02 mg/mL) to test the fungal growth. Blood samples were placed in the Bactec (BD Diagnostic Systems, Sparks, MD) system and daily checked for fungal growth.

The identification of the yeast isolates was accomplished by comparing the features of colonies on Brilliance *Candida* Agar (Oxoid) using the Vitek2 Compact (Biomerieux, France) following the manufacturer’s instruction. Molds were identified based on morphological characteristics with the help of commonly used keys. The strains isolated from the sterile samples were further reconfirmed by sequencing the internal transcribed spacer (ITS) regions, and the obtained nucleotides were compared to database sequences in GenBank [ 16 ].

**Deﬁnitions**

Fungal colonization (FC) was defined as the isolation of fungi from a non-sterile body site, while invasive fungal infection (IFI) was referred to the presence of fungal elements in sterile sites (wound biopsy or blood) [ 17 ]. Colonization index (CI) was defined as the ratio of the number of distinct non- sterile samples colonized by *Candida* to the total number of body sites cultured; accordingly, the patients with a CI of ≥ 0.5 were considered heavily colonized [ 18 ].

The adult (aged 16-65 years) who had the TBSA of ≥ 50% or FTBS of ≥ 10% were considered severely burned. The patients aged < 16 or > 65 years with a TBSA of ≥ 30% or an FTBS of ≥ 5% were defined as severe burn patients. All patients with respiratory burns or burns associated with multiple injuries were also considered to have a severe injury.

The diagnosis of renal failure was based on variations in creatinine and urine output according to the risk, injury, failure, loss of function, and end-stage renal disease criteria [ 19 ]. In addition, the patients with a fasting glucose level of > 126 mg/dl were considered to have hyperglycemia. The diagnosis of severe infection was performed based on the international guidelines for the treatment of sepsis and septic shock [ 20 ]. The patients who stayed in ICU for more than 14 days were regarded as having prolonged ICU stay [ 21 ].

**Statistical analysis**

The data were analyzed in SPSS software, version16.0. Continuous variables were reported by means and standard deviation and compared by Student’s t-test. Categorical variables were expressed as case number(n) and percentages and compared by the Chi-square test or Fisher’s exact test as appropriate. Univariate analysis (linear regression) was used to investigate the possible correlations of independent variables with dependent ones (FC or IFI). Factors with a p-value of <

0.05 in univariate analysis were included in the model of the multivariate analysis. Odds ratios (OR) and corresponding 95% conﬁdence intervals (95%CI) were calculated. Significance was set at a *p* value of 0.05 (two-tailed).

## Results

During the study time, there were 400 patients with a mean age of 29.7 years (range: 1-94) included in the research. The two most affected age groups were 1 ~ 10 (25%) and 31 ~ 40 (23.0%) years. Based on the obtained male: female ratio (3.5/1), males were found to be more affected than females. A total of 69 patients had inhalation injury, and 78 patients passed away during their ICU stay (Table 1). All infected patients were also colonized with fungi; accordingly, 360 (90%) patients were diagnosed with FC. Among 51 (12.25%) patients diagnosed with IFI, there were 3 patients with isolations from both blood culture and tissue biopsy (Table 2).

**Table 1:** Demographic characteristics of the patients

Characteristics		n	%
Age groups (years)	1-10	100	25.00
	11-20	34	8.50
	21-30	62	15.50
	31-40	92	23.00
	41-50	52	13.00
	51-60	31	7.75
	61-94	29	7.25
Mean of age ($\overset{-}{X}$ ±SD)		29.74±20.86	
Gender	Male	311	77.75
	Female	89	22.25
Severe infection		327	81.75
Severe burn		310	77.50
Hyperglycemia		171	42.75
Renal failure		81	20.25
Inhalation injury		69	17.25
APACHE>20		31	7.75
Urinary catheter		393	98.25
Prolonged ICU stay		369	92.25
Parenteral nutrition		300	75.00
Central venous catheters		276	69.00
Immunosuppressive therapy		210	52.50
Mechanical ventilation		124	31.00
Hemodialysis		59	14.75
Mortality		78	19.50

APACHE: acute physiology and chronic health evaluation

**Table 2:** Prevalence of fungal colonization/infection (n=400)

Fungal colonization/infection				n	Percentage (%)
Yes	FC			309	77.25
	Invasive fungal infection	FWI	Yeasts	22	5.50
			Molds	11	2.75
			Yeasts and molds	3	0.75
		Fungemia		12	3.00
		Fungemia+FWI		3	0.75
No				40	10.00

FC: fungal colonization, FWI: fungal wound infection

The frequency of fungal species isolated from burned patients is listed in Table 3. Among the 11 identified yeast species, *C. tropicalis*, *C. albicans*, and *C. parapsilosis* were the most prevalent ones, accounting for 94.6% of all isolates. Some representative ITS2 sequences of the isolates were submited to Genbank (*C. tropicalis* MN067757, *C. albicans* MT193530, *C. parapsilosis* MN067761, *C. glabrata* MN067758, *C. duobushaemulonis* MN174034, *Kodamaea ohmeri* MN067739, *A. oryzae* MH934917, *A. fumigatus* MH932411, *A. flavus* MN173209, *A. chevalieri* MN174037, *A. nomius* MN174036, and *F. solani* MN066126).

**Table 3:** Distribution of the isolated yeast species

Species	n	%	
		Overall (n=400)	Among yeast colonized (n=360)
*C. tropicalis*	164	41.00	45.56
*C. albicans*	151	37.75	41.94
*C. parapsilosis*	23	5.75	6.39
*C. lusitaniae*	4	1.00	1.11
*C. glabrata*	5	1.25	1.39
*C. dubliniensis*	4	1.00	1.11
*C. famata*	3	0.75	0.83
*C. ciferrii*	2	0.50	0.56
*C. krusei*	2	0.50	0.56
*C. tropicalis + C. duobushaemulonii*	1	0.25	0.28
*C. parapsilosis + K. ohmeri*	1	0.25	0.28

There were 28 patients colonized or infected with molds, with *Aspergillus* species as the predominant agent (27/28), followed by *Fusarium*. The most common *Aspergillus* species was *A. fumigatus* (39.29%; Table 4).

**Table 4:** Distribution of the isolated mold species

Species	n	%	
		Overall (n=400)	Among mold colonized (n=28)
*A. fumigatus*	11	2.75	39.29
*A. oryzae*	6	1.50	21.43
*A. flavus*	6	1.50	21.43
*A. chevalieri*	2	0.50	7.14
*A. nomius*	2	0.50	7.14
*F. solani*	1	0.25	3.57

The frequency of fungal species responsible for IFI is listed in Table 5. The most common agents of FWI were *Candida* (including *K. ohmeri*; n=26; 66.7% of FWI patients) and *Aspergillus* (n=15, 38.5%) species. *Candida tropicalis* was the most frequently isolated pathogen from the wound (n=17; 43.6% of FWI patients), followed by *C. albicans* (n=7; 17.95% of FWI patients) and *A. flavus* (n=6; 15.4% of FWI patients). Four patients were simultaneously overwhelmed by two different fungal species. Among three species of *Candida* causing *Candida*emia, *C. tropicalis* was the most prevalent species that accounted for 66.67% of the 15 yeast isolates. The other species were *C. albicans* (20.0%) and *C. parapsilosis* (14.33%).

**Table 5:** Distribution of the species causing invasive fungal infection

Species	Fungal wound infection (n=39)		Fungemia (n=15)	
	n	%	n	%
*C. tropicalis*	13	33.33	10	66.67
*C. albicans*	7	17.95	3	20.00
*C. parapsilosis*	1	2.56	2	13.33
*K. ohmeri*	1	2.56		
*A. fumigatus*	4	10.26		
*A. flavus*	4	10.26		
*A. oryzae*	2	5.13		
*A. chevalieri*	1	2.56		
*A. nomius*	1	2.56		
*F. solium*	1	2.56		
*C. tropicalis and C. duobushaemulonis*	1	2.56		
*C. tropicalis and A. fumigatus*	1	2.56		
*C. tropicalis and A. flavus*	2	5.13		

**Figure 1:**
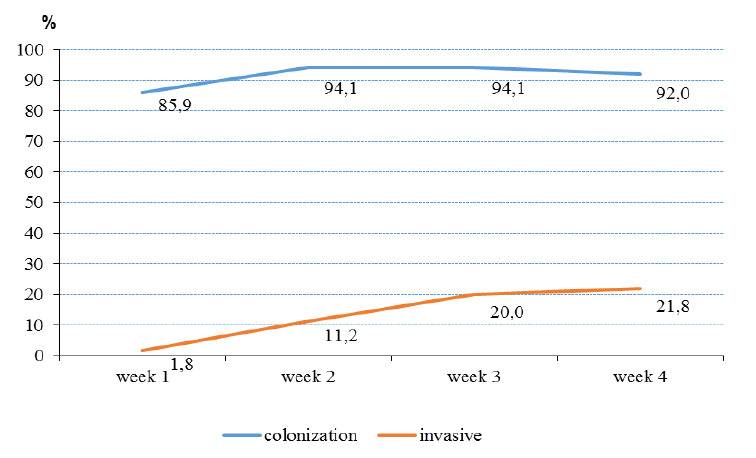
Prevalence of fungal colonization and infection in different weeks

The univariate analysis showed that severe infection, prolonged ICU stay, hyperglycemia, dialysis, and parenteral nutrition were significantly correlated with FC. However, in multivariate analysis, no factor was found to expose the patients to a higher risk of FC (Table 6). However, this analysis revealed many factors related to IFI. The results revealed hyperglycemia, prolonged ICU stay, and heavy *Candida* species colonization as the independent predictors of IFI (Table 7). Figure 1 shows the positive results of the specimens cultured within weekly intervals postburn. The frequency of IFI steadily increased during the stay in the hospital, while the prevalence of FC was nearly unchanged during the study period.

**Table 6:** Factors associated with fungal colonization

Variable	Univariate logistic regression		Multiple logistic regression	
	OR (95%CI)	*P*	OR (95%CI)	*P*
Severe injury	2.333 (1.207-4.512)	0.013	1.339 (0.629-2.852)	0.449
Inhalation injury	2.769 (0.829-9.252)	0.120		
Severe infection	2.407 (1.175-4.933)	0.028	1.620 (0.701-3.746)	0.259
Apache>20	3.545 (0.470-26.722)	0.088		
Prolonged ICU stay	1.512(0.781-2.926)	0.245		
Renal failure	2.449 (0.846-7.091)	0.061		
Hyperglycemia	2.818 (1.304-6.090)	0.004	1.355 (0.539-3.406)	0.518
Catheter	1.535 (0.785-3.004)	0.213		
Hemodialysis	7.645 (1.030-56.738)	0.018	4.758 (0.594-38.139)	0.142
Total parenteral nutrition	2.467 (1.258-4.836)	0.011	1.402 (0.626-3.138)	0.412
Mechanical ventilation	1.902 (0.850-4.256)	0.077		
Immunopressive therapy	1.753 (0.901-3.411)	0.099		

**Table 7:** Factors related to invasive fungal infection

Variable	Univariate logistic regression		Multiple logistic regression	
	OR (95%CI)	P	OR (95%CI)	P
Inhalation injury	1.576 (0.778-3.195)	0.233		
Severe injury	13.843 (3.311-57.885)	0.001	3.209 (0.626-16.458)	0.162
Burn shock	1.648 (0.376-7.229)	0.753		
Severe infection	12.996 (1.765-95.683)	0.001	0.255 (0.036-1.820)	0.173
Apache>20	1.350 (0.494-3.692)	0.573		
Prolonged ICU stay	4.520 (2.195-9.309)	0.001	2.611 (1.114-6.117)	0.027
Renal failure	3.729 (2.004-6.937)	0.001	1.101 (0.439-2.764)	0.837
Hyperglycemia	13.274 (5.509-31.982)	0.001	3.984 (1.309-12.129)	0.015
Catheter	8.491 (2.591-27.829)	0.001	0.951 (0.216-4.192)	0.947
Hemodialysis	6.723 (3.523-12.830)	0.001	2.071 (0.827-5.187)	0.120
TPN	19.800 (2.698-145.298)	0.001	4.724 (0.398-56.141)	0.219
Mechanical ventilation	3.875 (2.115-7.102)	0.001	0.696 (0.286-1.693)	0.424
Immunopressive therapy	8.364 (3.478-20.111)	0.001	2.418 (0.838-6.971)	0.102
Fungal colonization		0.005		0.997
Heavy fungal colonization	5.787 (2.406-13.920)	0.001	2.853 (1.095-7.437)	0.032

TPN: total parenteral nutrition

## Discussion

This study was targeted toward examining the prevalence and related factors of fungal colonization/ infection to provide guidance on the prevention and treatment practices of fungal infection for ICU burn patients. To the best of our knowledge, this is the first study that assessed the risk factors for fungal colonization/infection in burn patients residing in ICU in Vietnam. Our study revealed that ICU burn patients have some risk factors for IFI, which seemed to be remarkable for this set of vulnerable patients.

In the present study, the diagnosis of fungal colonization or infection was based on the detection of fungi in clinical samples by microscopic examination or culture. Microscopic potassium hydroxide preparation was used as it facilitates the visualization of fungal elements during direct microscopic examination. The H&E staining is the most common type of histopathological examination. Furthermore, PAS staining is useful for screening fungi in tissue sections as it binds with carbohydrate- rich macromolecules commonly found in fungal cell walls. Culture remains one of the key methods for diagnosing fungal infection, and in case of suspected fungemia, blood culture is currently considered the “gold standard” [ 22 ]. The other benefit of culture is the identification of the causative agents by conventional or molecular methods. The deﬁnite diagnosis of IFI, in the present study, was provided with histological and cultural evidence from biopsies or blood as required [ 23 ].

The results showed that males (77.75%) were more affected by burn injury, compared to females; moreover, the most common age group involved was 1-10 years. These findings are in concordance with previous reports in Vietnam [ 24 ], as well as China, a developing, neighbor country [ 25 , 26 ]. The mortality rate in the study was low (19.5%) and comparable to the value reported by Mundhada SG et al. (18%) [ 27 ], indicating the reducing trend of burn-related mortality in various regions [ 28 ].

Our findings showed that FC among the ICU burn patients was very common (90%), with 100% of the colonized patients having *Candida* isolation. The high rate of *Candida* colonization among those in ICU is in accordance with the data obtained by Caggiano et al. (2011) reporting the colonization of *Candida* species in 92.3% of the patients 15 days after ICU admission [ 29 ]. The very high rates of colonization by *Candida* species expose the patients to a high risk of IFI because the responsible agents for invasive candidiasis were mainly endogenous in origin [ 7 ], [ 30 ].

The FWI rate obtained in the present study is comparable to those reported by Capoor et al. (10%) [ 5 ], Gupta et al. (10%) [ 7 ], and Ali et al. (13%) [ 31 ]. In the current research, The prevalence of fungemia was 3.75%, which is in line with previous results [ 32 - 34 ]. This finding is indicative of the higher risk of fungemia in ICU burn patients, compared to that in the patients residing in other types of ICUs [ 35 , 36 ]. There is also a study reporting a higher incidence of fungemia in ICU burn patients (11%) [ 37 ].

In the present study, all colonized patients were found to be colonized with yeast species. This is comparable to previous reports describing the frequency of yeast (mostly *Candida* species) in burn patients [ 38 - 40 ]. It should be noted that the isolation rates of species belonging to CTG clade (*C. glabrata* and *C. krusei*) that were usually resistant to fluconazole were low (1.5% and 0.5%, respectively). This finding is in accordance with a previous study performed on Vietnamese patients [ 41 ]. In the present study, the results also revealed some other rare yeasts, such as *C. famata*, *C. duobushaemulonii*, and *Pichia (Kodamaea) ohmeri*. Those species have gained importance nowadays due to the emergence of nosocomial pathogens and their rapidly increased resistance to antifungal agents [ 42 - 46 ]. In line with the previous studies [ 40 , 47 ], the rate of colonization caused by mold was low (7%).

*Candida tropicalis* was the predominant agent responsible for both FWI and fungemia. In Vietnam, there are no prior data regarding the prevalence of IFI in ICU patients for comparative purpose. However, our results are in line with those of previous research with *C. tropicalis* being the most common agent causing *Candida*emia (50.5%) [ 41 ]. In concordance with the data reported in an Iranian study [ 31 ], in the present research, more than 40% of FWIs were caused by molds. The high rate of FWI caused by mold is disproportionate to the low prevalence of mold colonization among burn patients (7%). This signifies the importance of measures to protect the wound from mold colonization [ 48 ]. The source of mold infection is mostly exogenous when conidia as filamentous fungi fall onto the wound from the surrounding air and invade the wound immediately [ 8 ]. The most common mold was *Aspergillus* species (15/16 patients). This may demonstrate the strong virulence of *Aspergillus* among many other molds [ 49 ]. It is worth noting that there was an FWI case infected with *F. solani*. Although invasive infection caused by *Fusarium* is rare, it has caught much attention for its angioinvasive property, resistance to conventional antifungal therapy [ 50 ], and high mortality rate [ 51 ].

Consistent with other reports [ 29 , 52 , 53 ], the univariate analysis revealed severe infection, prolonged ICU stay, hyperglycemia, dialysis, and parenteral nutrition as the significant predictors of FC. However, based on the multivariate analysis, there were no factors predicting FC in burn patients. In our opinion, this is reasonable because most of the colonized patients had isolation upon the ICU admission, and the rate of FC was nearly steady over the period of the patients' ICU stay (Figure 1). Hedderwick et al. (2000) also found that most of the colonized patients were already colonized with yeast upon admission to the ICU, and only some of them became colonized after admission [ 54 ].

The results of our study showed that hyperglycemia, prolonged ICU stay, and heavy *Candida* species colonization were independently predictive of IFI. Hyperglycemia during acute hospitalization is a common pathophysiological phenomenon among severely burned patients [ 55 ]. Hyperglycemia is an adaptive response but could lead to some adverse outcomes, especially with increased risk of infections, including fungal infection [ 5 , 56 - 61 ]. In the current study, hyperglycemia was observed in 42.75% of the patients. Moreover, those with hyperglycemia were at 3.984 times higher (adjusted) risk of IFI, compared with those without hyperglycemia. Multiple site colonization is a well- known risk factor for invasive fungal infection and has been mentioned in many established *Candida* Prediction Rules [ 57 , 62 - 65 ]. In line with the results of previous studies [ 53 , 66 , 67 ], prolonged ICU stay was also found to be an important factor; in this regard, there was a clear rise in the infection rate from the second week (Figure 1) of hospital stay.

The other factors found to be related to IFI in univariate analysis included severe injury, severe infection, renal failure, hemodialysis, total parenteral nutrition, mechanical ventilation, catheter, and immunosuppressive therapy. However, these factors were not significant in multivariate analysis. Although these factors present in some *Candida* Prediction Rules, their impact may not be significant as they are absent in some other rules [ 57 , 62 - 65 ]. Therefore, their contribution as confounding factors in this study was rational. In the present research, high APACHE II score was not associated with the increased risk of IFI, which is in line with the findings reported by Escrig AIR et al. (2016) [ 68 ]. These findings are significant for clinicians to take timely precaution and prophylactic practices against fungal infections for high-risk patients.

## Conclusion

In conclusion, severely burned patients are at a risk of fungal infection mainly because of the pathophysiological consequences of injury and longer hospital stay. The most common agent was found to be *Candida* species (especially C. tropicalis), followed by *Aspergillus*. To ensure early and appropriate management measures, it is required to perform close monitoring on the presence of microbial agents in burn patients. It is also needed to well control blood glucose concentration in burn patients, especially in those staying in ICU for a long period.
